# Multi-Omic Analysis of Bacteriophage-Insensitive Mutants Reveals a Putative Role for the Rcs Two-Component Phosphorelay System in Phage Resistance Development in *Erwinia amylovora*

**DOI:** 10.3390/v17111487

**Published:** 2025-11-09

**Authors:** Nassereldin Ibrahim, Janet T. Lin, Darlene Nesbitt, Joshua Tang, Dharamdeo Singh, Lawrence D. Goodridge, Dion Lepp, Antonet M. Svircev, Joel T. Weadge, Hany Anany

**Affiliations:** 1 Guelph Research and Development Centre, Agriculture and Agri-Food Canada, Guelph, ON N1G 5C9, Canada; 2 Department of Biology, Wilfrid Laurier University, Waterloo, ON N2L 3C5, Canada; 3 Agriculture and Agri-Food Canada, London Research and Development Centre, Vineland Station, ON L0R 2E0, Canada; 4 Food Science Department, University of Guelph, Guelph, ON N1G 2W1, Canada

**Keywords:** fire blight, phage-carrier system, biopesticide, lytic phages, bacteriophage resistance

## Abstract

Phage therapy has garnered significant attention due to the rise of life-threatening multidrug-resistant pathogenic bacteria and the growing awareness of the transfer of resistance genes between pathogens. Considering this, phage therapy applications are being extended to target plant pathogenic bacteria, such as *Erwinia amylovora*, which causes fire blight in apple and pear orchards. Understanding the mechanisms involved in phage resistance is crucial for enhancing the effectiveness of phage therapy. Despite the challenges of naturally developing a bacteriophage-insensitive mutant (BIM) of *E. amylovora* (without traditional mutagenesis methods), this study successfully created a BIM against the podovirus ϕEa46-1-A1. The parent strain, *E. amylovora* D7, and the BIM B6-2 were extensively compared at genomic, transcriptomic, and phenotypic levels. The phenotypic comparison included the metabolic behavior, biofilm formation, and *in planta* evaluations of pathogenicity. The results revealed a mutation in strain B6-2 in the *rcsB* gene, which encodes a second regulator in the Rcs two-component phosphorelay system (TCS). This mutation resulted in significant changes in the B6-2 BIM, including downregulation of amylovoran gene expression (e.g., an average log_2_ fold change of −4.35 across *amsA-L*), visible alterations in biofilm formation, increased sensitivity to antibiotics (22.4% more sensitive to streptomycin), and a loss of pathogenicity as assessed in an apple seedling virulence model in comparison to the wildtype strain. The findings presented in this study highlight the critical role of the Rcs phosphorelay system in phage resistance in *E*. *amylovora*. Based on these findings, we have proposed a model that explains the effect of the B6-2 *rcsB* mutation on the Rcs phosphorelay system and its contribution to the development of phage resistance in *E. amylovora*.

## 1. Introduction

*Erwinia amylovora*, a Gram-negative bacterium, causes a serious disease known as fire blight in commercially grown apple (*Malus* X *domestica*) and pear (*Pyrus communis*) production [1,2]. According to Statistics Canada, economic losses in apples and pears were valued at an estimated USD 4 million in the 2005 outbreak (i.e., approximately 5% of the total production value), in addition to a substantial increase in production costs due to the spread of fire blight [3,4]. *E. amylovora* disease management in US and Canadian non-organic orchards relies mainly on antibiotics, but there are numerous accounts of how *E. amylovora* can form resistance to streptomycin, the main antibiotic used in management control practices of this pathogen [5,6]. AgriPhage^®^ is a commercial product registered in the US that uses a mixture of bacteriophages to control fire blight infections during open bloom. Researchers are also developing a phage-carrier system (PCS), which consists of *Pantoea agglomerans* cells that have been infected with known *Erwinia* phages [7,8]. In this PCS, *P. agglomerans* simultaneously acts as a phage delivery system (i.e., phage carrier and producer) and an antagonist biological colonizer to compete with *E. amylovora* [9,10]. A recently published protocol for the large-scale production and formulation of this PCS has been developed [11]. This study showed that a reconstituted PCS powder of *P. agglomerans* P39-7 infected with the ϕEa21-4 (myovirus, *Kolesnikvirus* Ea214) phage led to three log_10_ reductions in *E. amylovora* using a pear disc assay and a shelf life of the powder up to four months at 4 °C. However, to further leverage and enhance PCS efficacy, it is crucial to expand our current limited understanding of the phage resistance mechanism(s) of *E. amylovora* that can appear and how these may overlap with antibiotic resistance. Indeed, there is a co-evolving arms race between bacteria and their phages that involves bacterial resistance mechanisms and phage counter-defense strategies that can have wide-ranging consequences for the success of PCS products. Studying phage–bacterial dynamics has become important for many industries to succeed, including traditional cheese fermentation processes and the recent emergence of phage therapy applications to control plant, animal, and human pathogens [12,13].

In bacteria, two-component systems (TCSs) perceive various environmental signals, including phage–bacterial dynamics, to regulate cellular response to these cues [14,15]. For example, the Rcs (regulator of capsule synthesis) system is a non-orthodox TCS, first identified in *E. coli* and later shown to be conserved within the Enterobacteriaceae family, that plays a vital role in envelope stress response and virulence regulation [16]. Briefly, the Rcs system consists of a sensor outer membrane (OM) lipoprotein with kinase activity, RcsF, that is responsible for signal perception and Rcs activation [17]. RcsC is a transmembrane (TM) protein with histidine kinase/phosphatase activity, which acts as the first Rcs regulator protein that transfers phosphate groups from activated RcsF to RcsD, a second transmembrane (TM) protein in the system with phosphotransfer activity. The Rcs phosphorelay system is highly regulated, and in the absence of any environmental signal or membrane stress, phosphatase activities of both RcsC and RcsD ensure low-level phosphorylated RcsB [18]. RcsB is the second regulator protein with an output domain that binds upstream of target genes [19]. Upon activation of the Rcs phosphorelay system, RcsB is phosphorylated and the resultant conformational changes seem essential for homodimer or heterodimer (i.e., with RcsA or other auxiliary proteins) formation [19]. Importantly, dimer formation leads to binding to specific promoters/operons, which is absent in the monomeric form [20,21]. RcsB is considered a global regulator that can control the gene expression of various bacterial genes, including genes associated with capsule, cell wall and flagella biosynthesis, antibiotic resistance, biofilm formation, and virulence [22]. More specifically, in *E. amylovora* the Rcs system controls 648 genes [23], including those that play a pivotal role in the virulence and pathogenicity of *E. amylovora* in an immature pear fruit model [24].

The information on phage resistance in *E. amylovora* is sparse, partially due to the difficulty in generating *E. amylovora* phage-resistant mutants using the double agar overlay or liquid subculture methods [25]. The 1430^Y2R^ mutant is the only naturally developed resistant mutant to the Y2 phage reported in the literature [26]. In this mutant, the *Eamy_2231* gene encoding a putative galactosyltransferase is truncated, which is predicted to lead to inactive enzymes and loss of the phage receptor site; however, biochemical verification of the precise phage resistance mechanism/receptor is still lacking. This paucity of information on phage resistance mechanisms in *Erwinia* has hampered efforts to develop phage therapy measures as alternatives to antibiotics. Hence, the research presented herein reports a naturally developed bacteriophage-insensitive mutant (B6-2) that is resistant to the ϕEa46-1-A1 phage. The B6-2 mutant was extensively characterized across genomic, transcriptomic, and phenotypic levels to provide a broad understanding of the resistance development mechanisms and the impact on *E. amylovora* virulence. These results indicated that the main mutation is a two-amino-acid insertion in the product of the *rcsB* gene. Subsequent experiments provided evidence that the Rcs phosphorelay TCS plays a pivotal role in phage resistance development in *E. amylovora*. Furthermore, this phage resistance development was associated with increased susceptibility to streptomycin, a reduction in biofilm formation, and a loss of disease symptoms in infected plants when compared to the phage-sensitive parent strain. From these results, a working model for the effects of the *rcsB* mutation on the Rcs TCS has been proposed.

## 2. Material and Methods

### 2.1. Bacteria and Bacteriophage Isolates

All bacterial strains used in this study are listed in Table 1. Cultures were stored at −80 °C in Microbank cryobeads (Pro-Bank Diagnostics, Richmond Hill, ON, Canada). To prepare the working culture stock, one Microbank cryobead was mixed with one drop of phosphate-buffered saline (PBS) (10 mM, pH 7.2) (Sigma-Aldrich, Oakville, ON, Canada) and plated on 2.3% (*w*/*v*) Difco^TM^ nutrient agar (NA) plates (BD, Sparks, MD, USA). The plates were placed in an incubator (VWR, Avantor, Radnor, PA, USA) for 16 to 18 h at 27 °C and then stored at 4 °C for 1 to 2 wks. Working cultures were obtained from the initial cultures by streaking single colonies onto NA and incubating them at 27 °C for 16 to 18 h. Liquid cultures were then prepared from single colonies in Difco^TM^ nutrient broth (NB) medium (BD, Sparks, MD, USA) and incubated at 27 °C for 16 to 18 h.

The two lytic *E. amylovora* bacteriophages used in this study were ϕEa21-4 (myovirus) and ϕEa46-1-A1 (podovirus) as listed in Table 2. To propagate each phage, a bacterial host suspension was prepared by suspending 5–6 bacterial colonies in 3 mL of 0.8% (*w*/*v*) NB to obtain an OD_600_ of ~0.6. Using a 250 mL baffled Erlenmeyer flask, 100 μL of the bacterial suspension was added to 75 mL of NB, which was then placed in an incubator (New Brunswick Innova., Eppendorf, Hamburg, Germany) at 27 °C with 150 rpm shaking for 3 to 4 h. A 100 μL aliquot of phage stock (5.0 × 10^9^ plaque forming unit/mL (PFU/mL)) was added and the mixture was incubated for 16 to 18 h at 27 °C with 150 rpm shaking. Following incubation, 1 mL of chloroform (Sigma-Aldrich, Oakville, ON, Canada) was added to the culture and incubated with shaking for 5 min. The bacterial culture was subjected to centrifugation at 8500× *g* at 4 °C for 15 min in a Sorvall ST16R centrifuge (ThermoFisher Scientific, Waltham, MA, USA), the pellet discarded, and the supernatant filtered through a 0.22 μm Steriflip filter (Millipore, Burlington, MA, USA). The working phage stocks were stored at 4 °C in dark amber glass vials (Wheaton, DWK Life Sciences, Millville, NJ, USA) until needed. Bacterial and phage enumerations were carried out using a previously published quantitative PCR (qPCR) protocol [27].

### 2.2. Bacterial and Phage Enumeration by Plating

Bacterial cell cultures were serially diluted in PBS, and 100 μL of the dilutions were spread on NA and incubated for 16–18 h at 27 °C. Bacterial colonies were counted, and the final colony forming uint/mL (CFU/mL) was calculated accordingly by factoring in the dilution and plating factors and averaging the calculations of two replicate plates [29]. Phage plaques were counted by plating according to a previously published method [30]. Serially diluted phage solutions were spotted on a bacterial lawn, then plates were incubated at 27 °C for 16–18 h. Plaques were enumerated, and the final PFU/mL was calculated accordingly by factoring in the dilution and plating factors and averaging the calculations of two replicate plates.

### 2.3. Bacterial and Phage Enumeration by qPCR

Quantifying bacteria or phages by qPCR was carried out following a previously published protocol [27]. Briefly, a sample was first treated with DNase I to remove non-encapsulated genomes before qPCR. For qPCR, each 20 μL reaction consisted of 4 μL of MBI *EVOlution* 5 x Probe qPCR Mix (no ROX) (Montreal Biotech Inc., Dorval, QC, Canada), 200 nM of each primer, 100 mM of the specific bacteria/phage probe, and 2 μL of the template (i.e., bacterial or phage sample). The qPCR reactions were then carried out using a Bio-Rad CFX96 qPCR System (Bio-Rad, Hercules, CA, USA) that began with 10 min at 95 °C, followed by 40 cycles of 10 s at 95 °C and 45 s at 54 °C. A standard curve was generated by using three different dilutions (10^11^, 10^8^, and 10^5^ copies/mL) of the standard plasmid, pTotalStdA, which contains amplicons for *E. amylovora*, *P. agglomerans*, phage ϕEa21-4, and phage ϕEa46-1-A1. Using the generated standard curve, C_t_ (cycle threshold) values were used to calculate the corresponding genome copies/mL in unknown samples, assuming that each bacterial cell or phage capsid has a single genome.

### 2.4. Isolation of Bacteriophage-Insensitive Mutants (BIMs)

Two methods were tested for isolation of BIMs. Firstly, a double agar overlay method was used to isolate BIMs [31]. Bacterial cultures grown for 16–18 h in NB were inoculated with phages at different multiplicity of infection (MOI) values (1.0, 10, and 100). Then, infected cells were mixed with 4 mL soft agar, poured over 2.3% (*w*/*v*) NA and incubated at 27 °C for 24–48 h. Single bacterial colonies, representing possible BIMs, were cultured in NB and tested for phage sensitivity. Each BIM was subcultured four times and then challenged with phages to test the stability of phage resistance in the BIMs, followed by storage at −80 °C until needed. The second method for BIM isolation was a liquid subculture method that was previously described [25]. In this method, a culture of each strain in NB was infected by lytic phages at MOI values of 10, 100, and 1000. Cultures were then streaked onto NA plates after incubation for 16–18 h at 27 °C. Isolated colonies were selected and were purified by three consecutive subculturing steps on NA. Due to BIM instability, a modification to the liquid subculture method was introduced by adding 0.5 M NaCl to the NB medium with different MOI (10, 100, and 1000) values, then incubated at 27 °C for 45 h. Phage resistance was confirmed by challenging possible BIMs with phages and ensuring that the phage population did not increase in these BIMs.

### 2.5. Genome Sequencing and Variant Analysis

Frozen bacterial culture stocks stored at −80 °C were streaked onto NA plates, and single colonies were subcultured onto fresh NA plates. Three individual colonies were then used to inoculate NB cultures. Genomic DNA was extracted from three biologically independent cultures of both the wildtype and BIM strains using the Genomic DNA Purification Kit (Thermo Scientific™, Waltham, MA, USA), following the manufacturer’s instructions. Sequencing libraries were prepared from 1 ng of genomic DNA using the Nextera XT Kit (Illumina, San Diego, CA, USA). Each strain was prepared in triplicate and libraries were sequenced on a MiSeq instrument (Illumina) using a 600-cycle v3 reagent kit (Illumina, San Diego, CA, USA). The resulting 300 bp paired-end reads were quality-filtered with *Trimmomatic* (0.39) [32] to remove Nextera adapter sequences and low-quality bases using parameters “LEADING:30 TRAILING:30 SLIDINGWINDOW:4:30 MINLEN:200”. Single-nucleotide polymorphisms (SNPs) and insertion/deletions (INDELs) between the wildtype and BIM strains were identified with *GATK* (4.1.8.1) [33] based on GATK Best Practices. Briefly, reads were mapped to the (*Erwinia* Ea1189) reference genome with BWA-MEM (0.7.17-r1188) and duplicate reads identified with PicardTools (2.23.4-0) MarkDuplicates. Variants were called with the GATK HaplotypeCaller and sample genotypes assigned with GenotypeGCVFs. The resulting SNPs and INDELs were quality-filtered separately with GATK VariantFiltration using the parameters “-filter “QD < 2.0” -filter “QUAL < 40.0” -filter “SOR > 3.0” -filter “DP < 10” -filter “FS > 60.0” -filter “MQ < 40.0” -filter “MQRankSum < -12.5” -filter “ReadPosRankSum < -8.0”” and “-filter “QD < 2.0” -filter “QUAL < 40.0” -filter -filter “DP < 10” -filter “FS > 200.0” -filter “ReadPosRankSum < −20.0”, respectively. Variants were annotated with SNPEff (5.0.0) [34] and those conserved between replicate genomes were visualized and further inspected in Integrated Genome Browser (IGV; 2.8.9) [35].

### 2.6. RNA-Seq Analysis

Total RNA was isolated using the Ribominus^TM^ Bacteria Transcriptome Isolation Kit (Thermo Scientific, Waltham, MA, USA) as per the manufacturer’s recommendations. After RNA extraction from four biological replicates of both wildtype D7 and BIM strains, the purity of the isolated RNA was determined using a Nanodrop 2000 (Thermo Scientific™, Waltham, MA, USA) and quantified with a Qubit 2.0 using the RNA HS Assay Kit (Thermo Fisher Scientific, Ottawa, ON, Canada). RNA integrity was evaluated using a 2100 Bioanalyzer with the Agilent Technologies Agilent RNA 6000 Nano Assay Kit (Thermo Fisher Scientific, Ottawa, ON Canada). Samples with RNA integrity number (RIN) ≥ 8 were used for library construction. RNA was DNAase-treated and then assessed for residual DNA contamination by qPCR using *E. amylovora* levansucrase (*lsc*) species-specific primers and a no-reverse transcriptase control (NRT). The forward primer sequence is as follows: 5′-CGCTAACAGCAGATCGCA-3′. The reverse primer sequence is as follows: 5′-AAATACGCGCA-CGACCAT-3′, and the length of the PCR product is 105 bp [27].

RNA-seq libraries for sequencing were prepared using the Illumina Stranded Total RNA Prep, Ligation with Ribo-Zero Plus Microbiome kit (Illumina, San Diego, CA, USA) as per the manufacturer’s instructions (Illumina Document # 1000000124514 v03) with the exception of programming one less PCR cycle than that suggested during the library amplification. A total of 50 ng RNA input was used for rRNA depletion and cDNA library preparation. The cDNA library was quantified using a Qubit DNA HS Assay Kit (Thermo Scientific™, Waltham, MA, USA), and quality/size was evaluated using a 2100 Bioanalyzer with the Agilent Technologies High Sensitivity DNA Kit (Thermo Fisher Scientific, Ottawa, ON, Canada)Samples that showed adapter dimer formation at approximately 150 bp were further purified with AMPure XP beads (Beckman Coulter Life Sciences, Montreal, QC, Canada) using a 0.90X × bead tosample to reduce adapter dimer contamination in the sequencing run. The final library at 1 pM was sequenced using an Illumina NextSeq 500/550 High Output Kit v2.5 (150 Cycles) on an Illumina NextSeq 550. Quality assessment, mapping of filtered reads to the reference genome (*Erwinia amylovora* ATCC49946), and read counting were performed with the NextFlow nf-core RNASeq pipeline v 3.0 (https://doi.org/10.5281/zenodo.1400710, accessed on 26 April 2023) using STAR 2.6.1d for alignment and Salmon v1.4.0 for read counting. Differential gene expression (DEG) analysis was performed with the edgeR software v 3.36.0 [35] and visualized with degust v 3.1 [36,37]. Genes with an absolute log2 fold change (FC) > 2 and Log_10_ of the false discovery rate (FDR) > 100 were considered differentially expressed. Over-representation analysis (ORA) was performed on KEGG pathways and GO terms, and gene-set enrichment analysis (GSEA) was performed on KEGG pathways using the clusterProfiler v 4.2.2 package in R.

### 2.7. Phenotypic Comparison I: Phenotype MicroArray^TM^ (PM)

Metabolic comparisons between the parent strain and a BIM were evaluated globally using the Phenotype MicroArrays^TM^ (PM) system (Biolog Inc., Hayward, CA, USA), according to the manufacturer’s instructions. Using Biolog’s protocol, *E. amylovora* strains were cultured twice on BUG Agar with 5% (*v*/*v*) Blood plates (Biolog Inc., Hayward, CA, USA), and then colonies were suspended in PM IF-0a GN/GP Base inoculating fluid (Biolog Inc., Hayward, CA, USA) until the solution reached the required transmittance as determined by measuring turbidity by a turbidity meter (Cole-Parmer, Quebec, QC, Canada). After this, the Biolog Redox Dye Mix A (Biolog Inc., Hayward, CA, USA) was added to the culture as outlined in the standard protocol recommended by Biolog (Biolog Inc., Hayward, CA, USA) for *E. coli*. A total of 100 μL was added to each well in the Phenotype MicroArraysTM (PM) plates (https://www.biolog.com/products/metabolic-characterization-microplates/microbial-phenotype/, accessed on 27 October 2025) and incubated in an OmniLog incubator-reader (Biolog Inc., Hayward, CA, USA) at 27 °C for 48 h. Colorimetric data were collected every 15 min and analyzed using OmniLog PM software version 2.3.01 (Biolog, Hayward, CA, USA) [38] and using the DuctApe open-source package (http://combogenomics.github.io/DuctApe, accessed on 27 October 2025) [39].

### 2.8. Phenotypic Comparison II: Biofilm Formation

Both *E. amylovora* wildtype and B6-2 strains were inoculated in 10 mL NB, with 0.1 M NaCl (ThermoFisher Scientific, Ottawa, ON, Canada), and incubated without shaking for 7–10 d at 27 °C in the presence of type 316 stainless-steel coupons (Biosurface Technologies Corportaion, Bozeman, MT, USA) to induce biofilm structure. Coupons were processed for scanning electron microscope (SEM) imaging as previously described [40].

### 2.9. Phenotypic Comparison III: Pathogenicity

The “dirty scissors” method was used to test the pathogenicity of the B6-2 mutant [41]. Pear cuttings obtained from mature trees were inoculated by cutting the leaf edge along the midrib with scissors that have bacterial cell culture (10^9^ CFU/mL) on the blades. The positive control of *E. amylovora* D7 was used, while sodium phosphate buffer (Sigma-Aldrich, Oakville, ON, Canada) was used as a negative control. After 5 d of inoculation, blight symptoms were reported.

## 3. Results

### 3.1. Isolation of Bacteriophage-Insensitive Mutant (BIM)

Initial attempts to isolate *E. amylovora* BIMs using double agar overlay or liquid subculture methods were unsuccessful [31,42]. The resultant BIMs were transient (unstable) and were susceptible to phage infection after several subcultures. A modified liquid subculture protocol was therefore developed in this study, resulting in the isolation of one stable BIM from *E. amylovora* D7 (*Ea*D7) against the ϕEa46-1-A1 phage, hereafter named *E. amylovora* B6-2. Our developed protocol used a medium with salt (e.g., 0.5 M NaCl) and a high phage MOI of 1000, a combination that appears to have stressed/compromised *E. amylovora* enough to generate a BIM. Resistance to phage infection in strain B6-2 was confirmed by both spotting ϕEa46-1-A1 over a B6-2 bacterial lawn and by the use of ϕEa46-1-A1 primer-specific qPCR (Figure S1 and Table S1). Subsequent experiments were then carried out to compare the parent strain *Ea*D7 and the B6-2 BIM.

### 3.2. Comparative Genomics of E. amylovora D7 and B6-2 Strains

Comparing the genomic data of the *E. amylovora* D7 parent strain and the B6-2 BIM revealed consistent mutational differences (Table 3). Two mutations were found in all three of the BIM replicates. The first of these mutations included an insertion in *rcsB*, which is a component of the Rcs phosphorelay TCS [14]. Alignment of the predicted amino acid sequences of the wildtype RcsB with the mutant revealed a Leu101-Asp102 (L_101_D_102_) amino acid insertion (Figure S2A). Using the AlphaFold3 program, this insertion was predicted to be within the loop between the fourth α-helix and the fifth β-sheet of RcsB, which is predicted to lead to localized perturbations around this loop and possibly a more flexible C-terminus [43]. The second redundant mutation detected was a single-nucleotide polymorphism (SNP) in the aldehyde dehydrogenase gene *aldD*, found on the pEA29 plasmid, resulting in an Asp85 to Val85 (D_85_ > V_85_) amino acid substitution in the protein product (Figure S2B). Other non-redundant mutations (not present in all three BIM replicates) were noted in a DUF2931-domain containing protein gene, the electron transport complex gene *rsxC*, and phosphoenolpyruvate synthase gene *ppsA* (Table 3). 

### 3.3. Structural Modeling of the RcsB Mutant Using AlphaFold3

**Figure 1:**
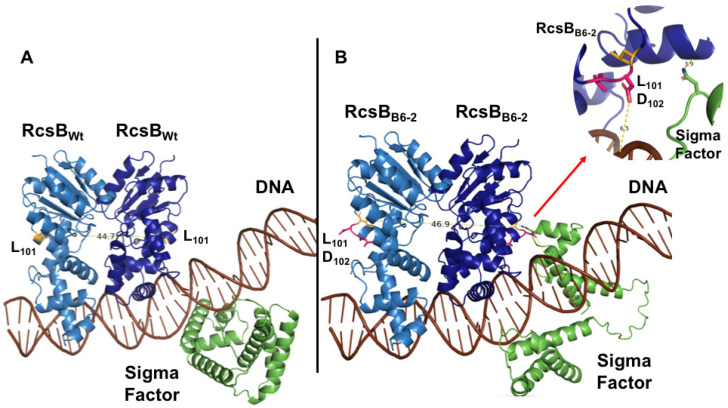
**The binding of the *E. amylovora* RcsB homodimer to an RcsAB DNA box in the presence of a sigma factor as predicted by AlphaFold3.** (**A**) Wildtype (accession #: A0A831EKB6) RcsB homodimer. (**B**) The B6-2 mutant RcsB homodimer. In both Panels A and B, the respective RcsB homodimer (blue) has also been modeled with the *E. amylovora* sigma factor (green) and the bound RcsAB DNA box (brown). The respective positions of L_101_ in the wildtype RcsB and in the mutant RcsB are shown in yellow, while the insertion of two new amino acids, a leucine and aspartate, are depicted in magenta in Panel B. The arrow in Panel B indicates a zoomed inset of the interaction between the RcsB mutant and the sigma factor with the adjacent DNA. The distance between the two L_101_ amino acids in the wildtype RcsB proteins of the homodimer is 44.7 Å, compared to 46.9 Å in the mutant RcsB homodimer. Structures were predicted with AlphaFold3 and imaged with PyMol Ver 1.8 [43,44].

In an attempt to understand the effect of the core *rcsB* mutation (i.e., the L_101_D_102_ insertion) on protein function, AlphFold3 was used to predict the conformational changes in RcsB structure as a homodimer that binds to an RcsAB DNA box (Figure S5) and also in the presence of an *E. amylovora* sigma factor (Figure 1) [43]. The predicted RcsB homodimer of the B6-2 mutant showed that the L_101_D_102_ amino acid insertion affects the compactness and spatial distribution of the secondary structure elements of the mutant RcsB homodimer near the insertion compared to that of the wildtype (Figure S5). This conformational change in the RcsB homodimer structure seems to drastically affect the binding of the sigma factor, during the formation of the transcription initiation complex, as predicted by AlphFold3 (Figure 1).

### 3.4. Comparative Transcriptomics of E. amylovora D7 and B6-2 Strains

To follow on the genomic and structural modeling results, comparative transcriptomics was performed to further delineate the importance of the Rcs system and to uncover other transcriptional effects that are occurring in the B6-2 BIM. Four replicate RNA samples were prepared from each of the *E. amylovora* D7 wildtype and B6-2 BIM strains and sequenced on an Illumina NextSeq instrument for transcriptomic analysis. The aligned sequence data from each sample showed greater than 15 M sequence reads uniquely mapped to the *E. amylovora* reference genome (Figure S3A), with greater than 13 M reads per sample mapping to protein-coding sequences and exon reads (Figure S3C,D). The B6-2 BIM samples clustered separately from the wildtype, as shown by the principal component analysis (PCA), indicating substantial differences in transcriptional profiles between the BIM and wildtype, which were consistent among replicates (Figure S3B). The overall quality control analysis of the transcriptomic data of the *E. amylovora* D7 and B6-2 BIM strains demonstrates the high quality of the data.

**Figure 2:**
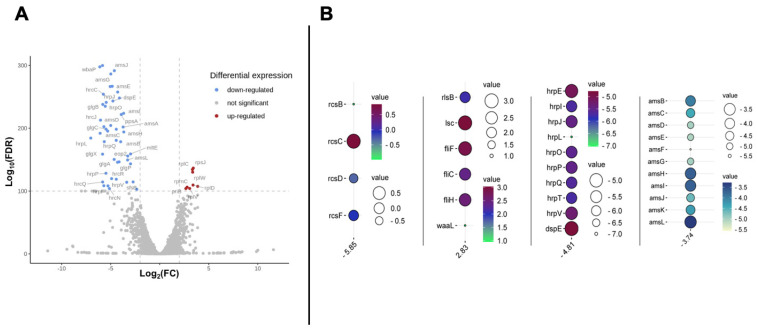
**Transcriptomic comparison between the *E. amylovora* D7 and B6-2 BIM strains.** (**A**) Volcano plot of differentially expressed genes. Significantly upregulated and downregulated genes (noted by Log_10_FDR < 100 changes) are represented as red and blue dots, respectively, on the plot. (**B**) A focused look at the differential expression of important genes from the Rcs system, levan biosynthesis, flagella, *hrp* genes, and *ams* operon. These data were generated by an online tool (http://www.bioinformatics.com.cn) where the color and size represent the Log_2_ FC values.

Given that the B6-2 BIM strain has an insertion mutation in the *rcsB* gene that leads to an insertion in a loop between the fourth α-helix and the fifth β-strand of the RcsB structure, the change is expected to directly affect genes that are regulated by the Rcs system and ultimately lead to the phage resistance. A volcano plot of the transcriptomic data of the B6-2 mutant versus the parent strain D7 showed downregulation of many genes in comparison to upregulated genes (Figure 2A). The significant differentially expressed genes (Log_10_False Discovery Rate (FDR) < 100) in the transcriptomic comparison are given in Table 2. This downregulation included key genes, such as the genes from the Rcs system, but also genes involved in amylovoran biosynthesis and hypersensitivity-regulation proteins (hrp). In contrast, key upregulation took place in the levan biosynthesis and flagella genes (Figure 2B).

The transcriptomic data showed strong downregulation of *rcsA*, along with other Rcs system gene members *rcsB*, *rcsD*, and *rcsF* (log_2_ fold change (FC) of −5.85, −0.99, −0.32, and −0.07, respectively) (Figure 2B and Table 4). However, in contrast to this trend, there was a slight upregulation in *rcsC* (log_2_ FC of 0.84). All 12 genes comprising the *ams* operon (*amsA-L*), which is responsible for amylovoran biosynthesis in *E. amylovora*, were downregulated, with an average log_2_ FC of −4.35. Similarly, drastic downregulation of *hrp* operon genes was observed, with an average log_2_ FC of −6.14. On the other hand, the transcriptomic data showed upregulation of levan biosynthesis genes (*rlsA*, *rlsB*, and *lsc* with log_2_ FC of 2.82, 2.10, and 3.03, respectively) and flagellar genes (*fliF*, *fliC*, and *fliH* with log_2_ FC of 2.89, 2.39, and 2.52, respectively) (Figure 2B and Table 2). Other genes, such as *hfq*, *cpxR*, and *cpxP*, were downregulated by Log_2_ FC of −0.94, −1.18, and −1.13, respectively. Also, *yiaD*, a putative lipoprotein from the OmpA-family was downregulated by Log_2_ FC of −4.29, while the putative retron Ec48 gene was upregulated by Log_2_ FC of −2.20.

### 3.5. Phenotypic Comparison of E. amylovora D7 and B6-2 Strains

#### 3.5.1. I: Phenotype MicroArray^TM^ (PM) Analyses

**Figure 3:**
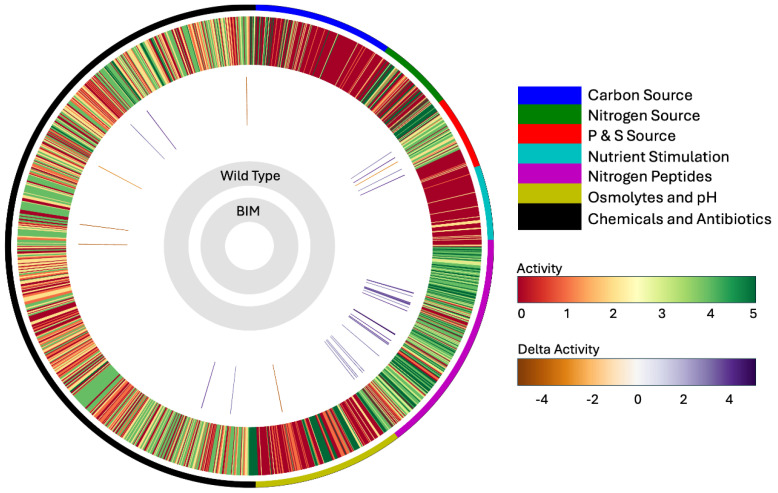
**Activity ring summary of the phenotype MicroArray data for the B62 BIM**. The outermost circle indicates the PM categories with a specific color for each category that corresponds to the legend noted in upper right of the figure. The PM activity for the wildtype *E. amylovora* D7 strain data is shown in the second outer circle with lines colored red to green according to an Activity Index (AV), scaled from 0 to 5 for low to high metabolic activity with a substrate, respectively. The differences in the PM activity between the B6-2 BIM compared to the parent D7 strain are shown as colored lines in the third outermost circle. Higher activity of the B6-2 BIM is indicated in black/purple, and the orange/brown color indicates a lower activity (noted as Delta Activity). These data depict a single replicate due to the scope of the analysis; however, separate trials with Biolog PM plates were also conducted on a smaller scale to ensure the high reproducibility of this process. This figure was generated with the DuctApe package [39].

**Figure 4:**
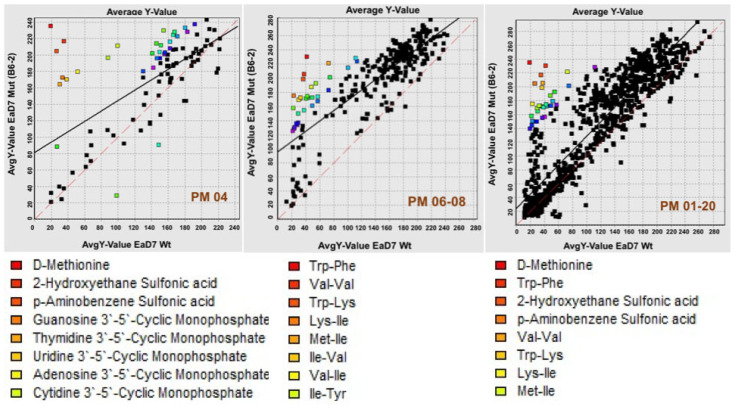
**Significant metabolic differences between the *E. amylovora* B6-2 BIM and D7 parent strains**. The Average Y-value data for PM04, PM06-08, and PM11-20 plates are shown as scatter plots. Well values are represented as squares, where the significant differences (*p* < 0.005) are displayed in colored squares. The data analysis was performed with the Biolog analysis software.

Phenotypic analysis was carried out using Biolog Phenotype MicroArray^TM^ (PM) plates with an OmniLog PM system. Phenotype MicroArray^TM^ plates include different chemicals that cover carbon-source (PM01-PM02), nitrogen-source (PM03), phosphorus- and sulfur-sources (PM04), nutrient stimulation (PM05), nitrogen peptides (PM06-08), osmolytes and pH (PM09-10), and chemicals (including antibiotics) (PM11-20). Growing the *E. amylovora* B6-2 mutant and D7 parent strains on the 20 PM plates gave metabolic activity differences between both strains (Figure 3). Significant differences in metabolic activities were observed in PM04 for the phosphorus and sulfur (P&S)-sources and in PM06-08 for the nitrogen peptides. Significant differences in antibiotic sensitivity were also observed in PM11-20. To express these differences in numbers, the Average Y-values (*p* < 0.005) were tabulated and presented as scatter plots (Figure 4). The metabolic differences are shown as a percentage (>20%) of gaining more activity (positive percentage values) or becoming more sensitive (negative percentage values) summarized in Table 5. One of these significant differences includes the increasing sensitivity to antibiotics, such as streptomycin, kanamycin, and tetracycline of B6-2 over the wildtype D7 strain. This is of particular importance since streptomycin is the antibiotic of choice in controlling fire blight.

#### 3.5.2. II: Biofilm Formation Analyses

**Figure 5:**
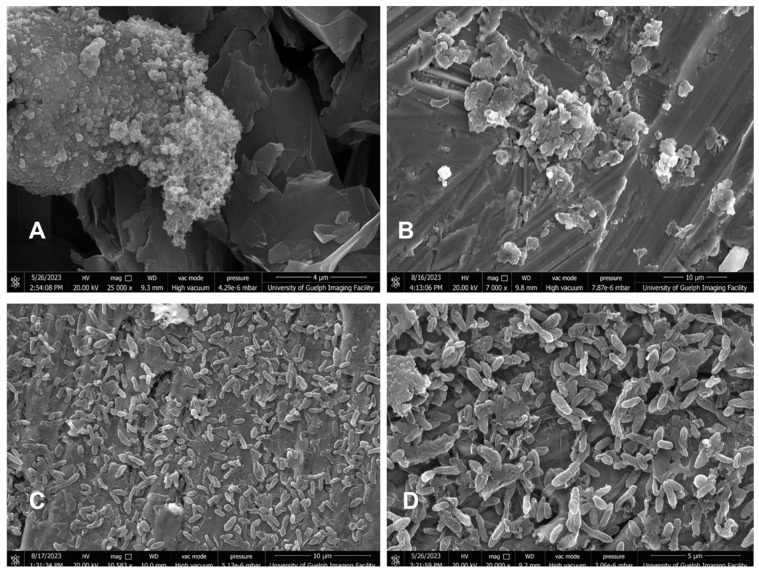
**Biofilm formation of *E. amylovora* D7 and the B6-2 BIM strains.** Both the *E. amylovora* D7 and B6-2 strains were inoculated into NB, with 0.1 M NaCl, without shaking for 7–10 d at 27 °C with stainless steel coupons to provide a surface for biofilm development. The experiment was repeated twice. (**A**,**B**) represent scanning electron microscopy images for the D7 strain and show evidence of the cell clumping biofilm structure. On the other hand, (**C**,**D**) represent scanning electron microscopy for strain B6-2 and show some aggregation or early-stage biofilm, but not the larger clumped structures evident for the D7 parent strain.

To further explore the differential regulation of amylovoran, flagellar, and levan biosynthesis genes (noted above in Figure 2 and Table 4), biofilms of the *E. amylovora* D7 and B6-2 strains were imaged by scanning electron microscopy. Cells were grown on stainless steel coupons placed in NB medium for 7–10 d at 27 °C before imaging. The wildtype D7 strain formed a biofilm with a clumped growth pattern of the cells on the coupon surface (Figure 5A,B). In contrast, the B6-2 BIM showed more diffuse cell aggregation consistent with an early biofilm stage that covered the surface under these same conditions (Figure 5C,D). However, there was no readily observable biofilm matrix associated with these aggregated cells by this method.

#### 3.5.3. III: In Planta Pathogenicity Model

**Figure 6:**
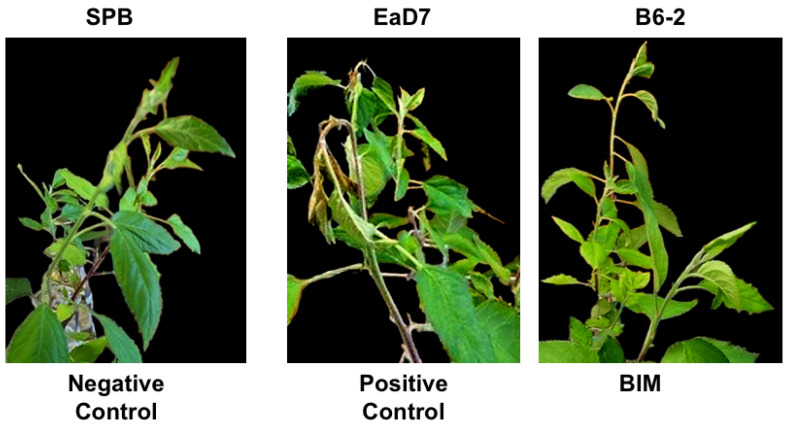
**Pathogenicity of *E. amylovora* D7 and the B6-2 BIM strains on apple seedlings.** Apple seedlings were inoculated with sodium phosphate buffer (SPB, negative control), *E. amylovora* D7 (positive control), and BIM B6-2 using the “dirty scissors” technique by dipping scissors into each solution at 10^9^ CFU/mL or SPB and snipping the primary leaf. After 5 d incubation, the negative control and the B6-2 inoculated seedlings showed no fire blight symptoms, while the wildtype *E. amylovora* D7 showed characteristic fire blight symptoms of shoot tip wilt and necrosis.

To study the effect of the B6-2 BIM mutation on pathogenicity, the “dirty scissors” method was employed [41]. Apple seedlings were inoculated by snipping the primary shoot leaf edge with scissors that had been dipped into a bacterial cell culture at 10^9^ CFU/mL, while sodium phosphate buffer was used as a negative control. Five days post-infection, the leaves inoculated with *E. amylovora* D7 showed blight symptoms (e.g., browning of leaves and shepherd’s crook at the end of the shoot); however, those inoculated with the B6-2 BIM, as well as sodium phosphate buffer (negative control), showed no symptoms (Figure 6).

In summary, the B6-2 mutant harbors an insertion mutation in the *rcsB* gene that leads to the insertion of two amino acids (LD). This mutation leads to a complete resistance to ϕEa46-1-A1, impaired amylovoran and T3SS biosynthesis, and an increase in flagellin and levan biosynthesis. These changes in the B6-2 BIM were accompanied by an increased utilization of nitrogen peptides during growth and increased sensitivity to streptomycin (the antibiotic of choice in treatment of *E. amylovora*). Finally, the B6-2 strain also displayed alterations in biofilm formation and a loss of pathogenicity in a plant model of infection.

## 4. Discussion

In the present work, an *E. amylovora* phage-resistant bacterial strain against the phage ϕEa46-1-A1 was developed naturally, without the assistance of transposon mutagenesis, using a modified liquid subculture method. This method involved using media with salt (0.5 M NaCl) and a high MOI value (1000) of phages, a combination that appears to have stressed/compromised *E. amylovora* enough to generate a BIM. No phage ϕEa46-1-A1 propagation was seen in this stable B6-2 mutant by either the standard plaque formation assay (Figure S1) or qPCR (Table S1). In an attempt to understand the mechanism(s) behind the phage resistance in this strain, a comprehensive comparison was performed between the B6-2 mutant and the wildtype *E. amylovora* D7 strain at the genomic, transcriptomic, and phenotypic levels.

Genomic analysis revealed many mutations in the B6-2 mutant compared to the D7 parent strain; however, only two mutations were present in all biological replicates. The first mutation was in the *aldD* gene, a pEA29 plasmid gene that encodes an aldehyde dehydrogenase family protein. The second mutation was in the *rcsB* gene, where there was a two-amino-acid insertion in a loop between the fourth α-helix and the fifth β-sheet of the AlphFold3-predicted RcsB structure (Figure S2). While both of these mutations were found in each of our isolates, our subsequent transcriptomic and phenotypic studies did not expand/clarify the role of the *aldD* mutation. In contrast, the importance of the *rcsB* mutation was readily observed in our subsequent corroborating analyses, so the effects of this mutation were able to be more fully developed/discussed. Indeed, given that RcsB is a global regulator, this was to be expected.

RcsB is the second regulatory protein in the Rcs TCS, which is known to be conserved within the family Enterobacteriaceae and plays a vital role in envelope stress response and virulence regulation [14,16]. The Rcs system is suggested to control 5% of bacterial genes and is considered a critical bacterial strategy for adapting to different environmental or internal signals [21]. These signals are broad and include changes in osmolarity, oxygen, sporulation, biofilm formation, nitrogen metabolism, cell wall integrity, virulence, antibiotic resistance, LPS, peptidoglycan, and membrane stress [45,46]. More than 648 genes in *E. amylovora* are reported to be controlled by the Rcs system, including many involved in virulence and pathogenicity [23].

Given the importance of the Rcs system, it was not surprising to find a drastic change in the transcriptomic data of the B6-2 mutant compared to the parent D7 strain, which supports the results of our observed genomic analysis. In the Rcs system (Figure S6), RcsF is the sensor kinase that is responsible for envelope and/or environmental signal perception and Rcs activation [13]. A phosphorelay of activation begins with this protein and then is passed on to the inside of the cell. RcsC possesses histidine kinase/phosphatase activities and can transfer phosphate groups from the activated RcsF to phosphotransfer protein, RcsD, and ultimately then on to RcsB, which is the output protein that leads to the regulation of Rcs-regulated genes [14]. Upon Rcs activation, RcsB becomes phosphorylated and forms a homodimer or heterodimer (either with RcsA or other auxiliary proteins), which in turn allows the dimers to bind to specific target promoter regions [18]. The *rcsA* gene was found to be strongly downregulated in the B6-2 BIM, which suggests impairment of the RcsA–RcsB heterodimer formation that would target the RcsAB/BB regulatory boxes in this strain. Similarly, *rcsB* and *rcsD* genes are downregulated but to a lower extent compared to that for *rcsA*, so RcsB homodimer formation and activation would also be impaired through transcriptional regulation. The only *rcs* gene that was found to be upregulated was *rcsC*, which is likely due to its phosphatase activity as a means for the cell to tightly control any activation of the Rcs system in the B6-2 mutant. Likewise, downstream regulation of all the RcsAB/BB boxes containing genes/operons would be affected, which is highlighted by the differential regulation noted in the rest of our transcriptomics analyses, including those that affect cell wall components and virulence (explained in more detail below).

In *E. amylovora*, amylovoran, T3SS, and LPS have all been identified as major virulence factors in *E. amylovora*, so the pairing of the emergence of phage resistance with the downregulation of these genes is particularly intriguing [47,48]. The *ams* operon has 12 *ams*-encoded proteins (AmsA-L) that participate in different stages of amylovoran biosynthesis [47]. The transcriptomics data indicated downregulation of all *ams* genes (Figure 2 and Table 2). Interestingly, other published data have shown parallel results, where mutational studies in *E. amylovora* showed that *rcsB*, *rcsC*, and *rcsD* mutants are non-pathogenic and an *rcsA* mutant had reduced virulence [24,49]. In these mutants, impairing the RCS phosphorelay system would lead to loss of both amylovoran synthesis and pathogenicity. This is consistent with the results presented here, where the *rcsB* mutation leads to downregulation of *rcsA, rcsB*, and *rcsF*, but upregulation of *rcsC*. Importantly, these changes also led, at least in part, to an increase in phage resistance that has not been noted previously.

In *E. amylovora*, *hrc* and *hrp* genes are clustered in a pathogenicity island and are known to regulate T3SS, an important virulence factor [48]. These T3SS genes are downregulated in the B6-2 mutant, along with many genes related to LPS-modifying enzymes (Figure 2 and Table 4). The pairing of the emergence of phage resistance in the B6-2 strain with the downregulation of these genes is particularly intriguing. In particular, the downregulation of the LPS genes may be leading to loss of proper LPS biosynthesis, which would clearly be related to phage resistance since LPS is a primary binding site for phages infecting Enterobacteriaceae. While the role in phage resistance for amylovoran (noted above) and T3SS genes may be less clear, they have previously been noted to have a clear effect on the virulence of *E. amylovora* [23,24]. The reduction/alteration in biofilm formation and loss of pathogenicity by the B6-2 mutant compared to the wildtype D7 strain provides additional indirect support for the role of these structures in virulence in our studies (Figure 5 and Figure 6, respectively).

In contrast, the transcriptomic data showed upregulation of flagella biosynthesis. In *E. amylovora*, as well as in *Salmonella enterica* and *Escherichia coli*, the Rcs system is a negative regulator for the *flhDC* operon, which encodes proteins involved in flagella biosynthesis. *E. amylovora* Rcs mutants showed reduced swarming motility on swarming plates [20,50]. By this principle, the downregulation of *rcsB* would clearly result in the upregulation of the flagellar operon and an increase in the flagellar biosynthesis noted in our transcriptomic data (Figure 2 and Table 4). The inversely regulated relationship between flagella and T3SS uncovered in our results was also reported in separate studies with *Pseudomonas aeruginosa*, where a strain lacking flagella showed increased expression of T3SS genes through upregulation of *exsA* (the master regulator for T3SS genes) [51]. While these results further emphasize the importance of the Rcs phosphorelay system, they also have other implications for flagellar biosynthesis in phage ϕEa46-1-A1 resistance. Our transcriptomic data suggest that the upregulation of flagella did not lead to increased infectivity by the phage. Thus, it is possible that the flagella synthesized under these conditions was not recognized/utilized productively by the phage, or the phage does not use this as a receptor at all.

The B6-2 transcriptomic data also showed upregulation of levan biosynthesis genes. Levan is an EPS secreted from many Gram-negative bacteria, and activators for the expression of the *lsc* (levansucrase) gene are *rlsA*, *rlsB*, and *rlsC* [51,52,53]. In the transcriptomic data, all the *lsc*, *rls*A, *rls*B, and *rlsC* genes are upregulated. The Rcs system is known to control levan biosynthesis in *E. coli*, as well as in *Erwinia stewartii* through *rcsA* [54,55]. However, in *E. amylovora*, it was reported that overexpression of *rcsB* resulted in increased amylovoran production and suppressed expression of levansucrase, the key enzyme for levan biosynthesis, while the *rcsA* mutant showed reduced production of both amylovoran and levan [49,56]. This work emphasizes the difference in promoter/operon targets between the RcsB homodimer (RcsB-RcsB) and heterodimer (RcsA-RcsB) complexes and is consistent with our combined transcriptional and genetic data for an *rcsB* mutant.

To further explore the combined upregulation of flagellar and levan biosynthesis in conjunction with the downregulation of *rcs* genes, amylovoran, T3SS, and LPS, a microscopic examination of the cells was employed. Based on the results of these scanning electron microscope images, these biosynthetic processes (and their products) may be leading to a diffuse biofilm pattern observed for the B6-2 BIM cells (Figure 5), since extracellular polysaccharides (levan) and flagella often have morphological effects on community structure. In contrast, the elevated production of amylovoran and T3SS by the wildtype D7 strain could be factors inherently responsible for the clumping morphotype and biofilms that were observed for these cells. These results provide a foundation for using electron microscopy as a confirmatory method for further genetic/transcriptomic work in *E. amylovora* and could be a facile way to infer implications for future phage research applications on different surfaces.

There are a number of other differentially regulated genes in the B6-2 mutant that are likely to have direct effects on the virulence and phage resistance of this strain. For example, RprA is an Hfq-dependent small RNA (sRNA) that is known to affect the virulence of *E. amylovora*, and its expression is regulated by the Rcs phosphorelay system [57]. The B6-2 transcriptomic data showed downregulation in the *hfq* gene that encodes the RNA chaperone that is very important for RprA function. Additionally, the Cpx TCS (responsible for envelope stress and antibiotic sensitivity) seems to be affected by the *rcsB* mutation as the transcriptomic data showed downregulation of *cpxR* and *cpxP* genes, and that might explain the observed increased sensitivity to streptomycin in B6-2, as changes in Rcs and Cpx TCSs are able to modify the antibiotic sensitivity [58]. These results strongly highlight the role of the Rcs system in resistance development in the BIM and its interactions with other two-component systems that may have even broader implications for the cell.

Interestingly, the transcriptomic data also revealed an increase in gene expression of a putative retron Ec48 (accession #: WP_223473354). Retrons are bacterial genetic elements consisting of a reverse transcriptase and non-coding RNA (ncRNA) that act to protect against phage infection via abortive infection [59,60]. Retrons help to remove the individual infected cells from a larger population and eliminate successive phage infection (i.e., abortive infection). Thus, it is plausible that this system is also active as a secondary method (in addition to the Rcs system changes) that are aiding in resistance in B6-2 cells. It is worth mentioning that OmpA, which represents the most abundant outer membrane protein in Gram-negative bacteria, is believed to be a phage receptor for *E. amylovora* phages (ϕEa21-4 and ϕEa46-1-A1) [61]. Transcriptomic data showed strong downregulation of the *yiaD* gene, which encodes a putative OmpA-like protein (Table 4) [62]. Thus, it is plausible that the loss of YiaD in B6-2 may be another mechanism of phage resistance.

In follow-up work with the phenotypic microarray data, major differences were noted in the B6-2 BIM utilization of P&S-sources, nitrogen peptides, and antibiotic sensitivity (Figure 3 and Figure 4 and Table 5). Interestingly, the pathogenicity and hypersensitive response genes, *hrp*, are regulated by the positive regulator, *hrpL* in *E. amylovora* [63]. The nutritional environment, including carbon and nitrogen sources, affects the transcription of the *hrp* genes, where these genes are repressed in a rich medium (e.g., Luria broth). The results of the transcriptomic data in this study confirmed this relationship through the downregulation of *hrp* genes in the B6-2 strain (also grown in rich media). Thus, the phenotypic data support the metabolic changes predicted using the transcriptomics data (Figure S4). Additionally, the phenotypic microarray data highlighted key increases in antibiotic sensitivity of the B6-2 mutant (Table 5) to a number of antibiotics. The most important of these changes was the fact that the B6-2 mutant is more sensitive to streptomycin, which is the antibiotic of choice to treat *E. amylovora* and control fire blight.

**Figure 7:**
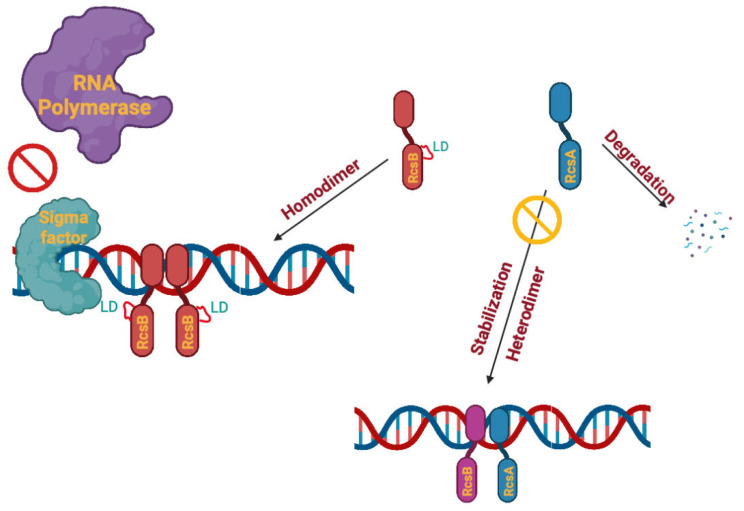
**A model for the effect of the *rcsB* mutation on the *E. amylovora* Rcs phosphorelay system.** In the inactivated/default state of the Rcs system (i.e., no stress signals), RcsA binds to non-phosphorylated RcsB to form a heterodimer that activates specific gene targets, including the *rcsA* gene itself. In the presence of environmental stress, RcsF receives the signal and ultimately induces RcsB phosphorylation, where it binds to an RcsAB DNA box as a homodimer instead. This will lead to downregulation of the *rcsA* gene and induce RcsA degradation^18^. In the B6-2 BIM, insertion of LD amino acids in the loop between a-helix 4 and b-strand (a4–b5) leads to conformational changes that resemble the RcsB phosphorylation upon Rcs system activation. Consequently, RcsB forms a homodimer that binds to an RcsAB DNA box. These conformational changes alter sigma factor binding and/or impair the formation of a proper transcription initiation complex and ultimately inhibit transcription.

The results presented herein provide key information on the role of the Rcs phosphorelay system in phage resistance development and pathogenicity of the *E. amylovora* B6-2 mutant. Previous studies have shown that *rcsB* plays a critical role in amylovoran production and phage susceptibility in *E. amylovora*. In particular, Roach et al. (2013) demonstrated that an *rcsB* knockout mutant of *E. amylovora* strain D7 produced less than 1% of the wildtype level of amylovoran, as quantified using cetylpyridinium chloride [64]. Additionally, the efficiency of plating of podovirus ϕEa46-1-A2 on the *rcsB* mutant was reduced to 0.0003 compared to the wildtype. These results have shown that amylovoran is essential for podovirus infection in *E. amylovora*. Hence, the following model of the Rcs system has been proposed to explain these concurrent phenomena. RcsB is at the center of this model based on the insertion of the two new LD amino acids into RcsB, which leads to the new sequence of -L_101_D_102_L_103_D_104_L_105_D_106_- in the loop between α-helix 4 and β-strand 5. Consequently, this increase in negative charge density due to the local presence of three negative aspartyl (D) residues may lead to conformational changes in RcsB that resemble the phosphorylation of RcsB during regular Rcs system activation. These conformational changes in RcsB make the formation of the homodimer (RcsB–RcsB) more preferable than the heterodimer (RcsB–RcsA). The homodimer preference in turn leads to RcsA degradation, as well as downregulation of *rcsA* expression that requires the heterodimer to be activated (Figure 7) [14,18]. Given that the conformational changes in RcsB resemble activation of the Rcs system (i.e., as would normally be seen upon environmental stress detection and subsequent phosphorylation of RcsB), it is conceivable that the mutant RcsB is able to alter sigma factor binding in the RcsB-RcsB form and affect formation of the transcriptional initiation complex, as predicted by AlphFold3 (Figure 7). The predicted RcsB homodimer alterations specifically affect the compactness and spatial distribution of the secondary structure elements of the mutant RcsB dimer compared with that of the wildtype. Thus, regulation of the transcription of these genes is impaired and genes requiring a proper complex for transcription activation would be noted as downregulated in our transcriptomic data of the B6-2 mutant.

This proposed model is supported in part by previously published data. A prime example of this setup has been seen with the *E. coli* phosphate regulon PhoB (Asp53Ala/Tyr102Cys) mutant. In this mutant, PhoB is homodimerized and activates the PhoB regulon, bypassing the phosphorylation requirement in spite of the fact that the PhoB mutant cannot be phosphorylated due to an Asp53Ala mutation [65]. The authors thus assumed that the Tyr102Cys substitution leads to substantial displacement of α-helix 4 towards the active site cavity, and these conformational changes lead to the formation of an activated protein that bypasses the requirement for phosphorylation. While these parallels are apparent, confirmation of the proposed RcsB model will require further biochemical analysis of the RcsB mutant. Regardless, the present model fully accounts for the downstream transcriptional downregulation of genes that are affected by RcsA–RcsB activation (EPS amylovoran, LPS and T3SS) and play a role in phage resistance and pathogenicity.

In summary, the combination of the genotypic, transcriptomic, and phenotypic results presented herein suggest that phage resistance in *E. amylovora* may be a multipronged approach that is affected by (1) the Rcs phosphorelay system (i.e., LPS, biofilm, and T3SS changes), as well as (2) an abortive infection process (retron EC48) and/or (3) an OmpA/YiaD-related mechanism. Importantly, the changes in the Rcs phosphorelay system not only affect phage resistance, but also result in loss of pathogenicity in the plant model. Whether directly connected or by a serendipitous effect, these cells are also more susceptible to streptomycin. These changes appear to be a magic bullet for the treatment of *E. amylovora*, since either the phage infects and kills the bacteria or traditional antibiotics (like streptomycin) are made more effective if resistance arises. Combined, these results provide a foundation for further work to develop phage- and/or antibiotic-based control methods of *E. amylovora* and fire blight.

## Figures and Tables

**Table 1:** *E. amylovora* strains used in this study.

Strain	NCBI GenBank Accession (Number)	Ref.
EaD7	JAAEUT000000000	[10]
B6-2	-	This work
Ea6-4	JAAEVD000000000	[10]

**Table 2:** *E. amylovora* phages used in this study.

Phage	Species	NCBI GenBank Accession Number	*E. amylovora* Host	Ref.
ϕEa21-4	*Kolesnikvirus* Ea214	NC_011811.1	Ea6-4	[28]
ϕEa46-1-A1	-	N/V	EaD7	[28]

**Table 3:** Mutations detected in *E. amylovora* B6-2 BIM replicates.

Chromosome (Position)	Change	Direct effect	Redundance	Gene	Protein ID	Product	Comments
NZ_CP055227.2 (1,361,368)	AAGATCC	Insertion L_101_D_102_	All	*rcsB*	WP_004158545.1	transcriptional regulator	
NZ_CP055228.1 (6011)	A to T	Missense D_85_ > V_85_	All	*aldD*	WP_013036331.1	aldehyde dehydrogenase family protein	pEA29 plasmid
NZ_CP055227.2 (617,179)	A to G		Two	-	WP_199266228.1	DUF2931 protein	Misassembled
NZ_CP055227.2 (617,202)	C to A		One	-	WP_199266228.1	DUF2931 protein	Misassembled
NZ_CP055227.2 (617,206)	A to T		One	-	WP_199266228.1	DUF2931 protein	Misassembled
NZ_CP055227.2 (617,207)	C to A		One	-	WP_199266228.1	DUF2931 protein	Misassembled
NZ_CP055227.2 (617,209)	C to G		One	-	WP_199266228.1	DUF2931 protein	Misassembled
NZ_CP055227.2 (1,982,326)	Inser. 241 nt		One	*rsxC*	WP_199266235.1	ETC complex subunit	Misassembled
NZ_CP055227.2 (2,029,451)	G to A		One	*ppsA*	WP_004157400.1	PEP synthase	

**Table 4:** Abbreviated list of significant differentially (Log_10_FDR < 100) expressed genes in *E. amylovora* B6-2 BIM replicates.

Gene	Product	FDR *	Log_2_ FC **	Gene	Product	FDR	Log_2_ FC
*amsA*	Amylovoran biosynthesis	1.23E-202	−3.74	*hrpB*	T3SS	1.00E-100	−6.61
*amsB*	Amylovoran biosynthesis	3.06E-179	−3.98	*hrpF*	T3SS	0.00E+00	−7.63
*amsC*	Amylovoran biosynthesis	3.78E-199	−4.43	*hrpG*	T3SS	1.00E-100	−6.51
*amsD*	Amylovoran biosynthesis	4.15E-205	−5.00	*hrpI*	T3SS	2.92E-109	−5.70
*amsE*	Amylovoran biosynthesis	3.84E-267	−5.05	*hrpJ*	T3SS	6.61E-242	−5.46
*amsF*	Amylovoran biosynthesis	1.00E-100	−5.56	*hrpL*	Sigma factor HrpL	4.58E-185	−7.03
*amsG*	Amylovoran biosynthesis	3.64E-287	−4.99	*hrpN*	Harpin	1.00E-100	−6.92
*amsH*	Amylovoran biosynthesis	8.81E-195	−3.66	*hrpO*	T3SS	1.00E-235	−5.55
*amsI*	Amylovoran biosynthesis	1.57E-224	−3.68	*hrpP*	T3SS	5.03E-129	−5.48
*amsJ*	Amylovoran biosynthesis	2.24E-292	−4.63	*hrpQ*	T3SS	2.13E-179	−5.65
*amsK*	Amylovoran biosynthesis	0.00E+00	−4.29	*hrpV*	T3SS	5.04E-109	−5.28
*amsL*	Amylovoran biosynthesis	3.60E-150	−3.28	*hrpW*	Put. pectate lyase	1.00E-100	−7.98
*dspE*	T3 effector protein	4.21E-244	−4.78	*mltE*	murein transglycosylase E	9.43E-160	−2.97
*eop2*	T3 effector (pectin lyase)	1.79E-156	−3.26	*ppsA*	PEP synthase	1.35E-222	−3.92
*glgA*	Glycogen synthase	4.08E-151	−4.65	*priB*	Primosomal replicat. protein N	1.08E-104	2.67
*glgB*	Glucan branching enzyme	1.06E-238	−5.81	*rcsA*	Colonic capsular biosynth. A. P.	1.00E-100	−5.85
*glgC*	G-1-P adenylyltransferase	3.15E-199	−5.47	*rplC*	50S ribosomal protein L3	4.28E-135	3.34
*glgP*	Glycogen phosphorylase	1.34E-146	−4.30	*rplD*	50S ribosomal protein L4	2.67E-110	3.40
*glgX*	Glycogen debranching enzyme	1.70E-159	−5.83	*rplV*	50S ribosomal protein L22	8.63E-105	3.06
*hrcC*	T3SS	7.03E-255	−5.75	*rplW*	50S ribosomal protein L23	8.17E-131	3.36
*hrcJ*	T3SS	1.25E-213	−6.04	*rpmC*	50S ribosomal protein L29	6.49E-107	2.80
*hrcN*	T3SS	1.06E-104	−5.13	*rpsJ*	30S ribosomal protein S10	2.73E-137	3.42
*hrcQ*	T3SS	3.76E-115	−5.82	*sfsB*	Sugar ferment. stimulation protein	1.03E-114	−3.37
*hrcR*	T3SS	1.49E-120	−4.91	*wbaP*	UDP-Gal::UDP transferase	1.81E-298	−6.11
*hrpA*	T3SS	1.00E-100	−7.60	*yiaD*	Putative OmpA-family	2.42E-258	−4.29

* FDR: false discovery rate. **: Log2 FC: log2 fold change.

**Table 5:** Metabolic gained/lost changes in the *E. amylovora* B6-2 BIM.

Metabolic Activity Gained or Sensitivity Lost				
Plate Type	Wells	Chemical	Difference *	Info
PM09	A09	7% NaCl	46.05	Osmotic sensitivity, NaCl
PM09	C10	6% NaCl + TMA	44.81	Osmolyte, trimethylamine (TMA)
PM12B	A11	Carbenicillin	41.20	Cell wall, lactam
PM13B	B05	2,2′- Dipyridyl	40.39	Chelator, lipophilic
PM09	F11	11% Sodium Lactate	39.86	Osmotic sensitivity, sodium lactate
PM09	B09	6%NaCl + Phosphorylcholine	38.73	Osmolyte, phosphorylcholine
PM11C	C10	Minocycline	35.02	Protein synthesis, tetracycline
PM09	C05	6%NaCl + g-Amino-Butyric acid	34.04	Osmolyte, g-amino butyric acid
PM09	B03	6%NaCl + Dimethyl Glycine	33.17	Osmolyte, dimethylglycine
PM09	C01	6% NaCl + KCl	32.28	Osmolyte, KCl
PM09	C04	6% NaCl + b-Glutamic acid	30.64	Osmolyte, b-glutamate
PM16A	E12	Rifamycin SV	28.71	RNA polymerase
PM09	B02	6% NaCl + Betaine	27.19	Osmolyte, betaine
PM19	G12	Hydroxylamine	25.58	DNA damage, antifolate
PM18C	E01	Sodium metasilicate	22.98	Toxic anion
PM09	B01	6% NaCl	22.79	Osmotic sensitivity, NaCl control
PM10	A04	pH 5	22.03	pH, growth at 5
**Metabolic Activity Lost or Sensitivity Gained**				
**Plate Type**	**Wells**	**Chemical**	Difference *	**Info**
PM10	D01	pH 4.5 + Anthranilic Acid	−108.7	pH, decarboxylase
PM13B	G05, G06	Manganese Chloride	−66.8	toxic cation
PM09	A12	10% NaCl	−60.9	osmotic sensitivity, NaCl
PM12B	H05, H06	Rifampicin	−54.0	RNA polymerase
PM12B	A07	Tetracycline	−51.4	protein synthesis, tetracycline
PM13B	H09, H10	Tylosin tartrate	−48.8	protein synthesis, macrolide
PM09	D12	20% Ethylene Glycol	−46.8	osmotic sensitivity, ethylene glycol
PM09	B07	6% NaCl + Ectoine	−43.1	osmolyte, ectoine
PM16A	H12	Sorbic Acid	−41.4	respiration, ionophore, H+
PM09	A05	5% NaCl	−40.5	osmotic sensitivity, NaCl
PM13B	D10	Rolitetracycline	−37.9	protein synthesis, tetracycline
PM09	C06	6% NaCl + Glutathione	−37.3	osmolyte, glutathione
PM09	H04	60mM Sodium Nitrate	−33.1	toxicity, nitrate
PM09	A10	8% NaCl	−32.3	osmotic sensitivity, NaCl
PM17A	E06	Compound 48/80	−29.6	cAMP phosphodiesterase inhibitor
PM09	C03	NaCl + N-Acetyl-L-Gln	−29.5	osmolyte, acetyl glutamine
PM09	B10	NaCl/Creatine	−29.4	osmolyte, creatine
PM12B	D04	Sisomicin	−29.0	protein synthesis, aminoglycoside
PM09	D01	3% Potassium Chloride	−27.7	osmotic sensitivity, KCl
PM09	B06	6% NaCl + MOPS	−25.8	osmolyte, MOPS
PM13B	E12	Ruthenium Red	−25.3	respiration, mitochondrial Ca^2+^ porter
PM15B	H12	Zinc Chloride	−25.0	toxic cation
PM09	H06	Sodium Nitrate	−24.7	toxicity, nitrate
PM12B	H09	Dodecyl TMAB **	−24.1	membrane, detergent, cationic
PM12B	H01	Spiramycin	−23.1	protein synthesis, macrolide
PM20B	H06	Tolylfluanid	−22.9	fungicide, phenylsulphamide
PM16A	E04	Streptomycin	−22.4	protein synthesis, aminoglycoside
PM10	F07	pH 9.5 + L-Tyrosine	−21.2	pH, deaminase
PM13B	H05	Moxalactam	−21.0	wall, lactam
PM10	G06	pH 9.5 + Putrescine	−20.8	pH, deaminase
PM09	E06	6% Sodium Formate	−20.6	osmotic sensitivity, sodium formate
PM18C	H10	Plumbagin	−20.5	oxidizing agent
PM11C	H10	Ofloxacin	−20.4	DNA topoisomerase
PM16A	A03	Cefotaxime	−20.3	wall, cephalosporin
PM11C	H05	Kanamycin	−20.1	protein synthesis, aminoglycoside
PM09	H07	10 mM Sodium Nitrite	−20.0	toxicity, nitrite

* Difference in activity/sensitivity when compared to the wildtype *E. amylovora* D7 strain. ** TMAB: Trimethyl Ammonium Bromide.

## Data Availability

The datasets used and/or analyzed during the current study are available from the corresponding author on reasonable request. All sequencing data generated in this study have been deposited in NCBI GenBank under Bioproject accession number PRJNA1232958 for genome sequencing of the Erwinia strains used in the study and under GEO accession number GSE291879 for the RNA-seq data.

## Supplementary Materials

The following supporting information can be downloaded at: https://www.mdpi.com/article/10.3390/v17111487/s1, Figure S1: Soft agar overlay with BIM 6-2 as host and spot tests with 6 *Erwinia* phages (clockwise) φEa9-2, φEa46-1A2 (Svircev), φEa35-70, φEa21-4, φEa46-1A1(Svircev) and φEa46-1A2 (G); Figure S2: Predicted structural modification caused by the E. amylovora BIM B6-2 mutations; Figure S3: Quality Control Analysis of the Transcriptomic Data of the E. amylovora Strains; Figure S4: Metabolic Changes Due to the Transcriptomic Data of the E. amylovora Strains; Figure S5: Conformational changes in RcsB homodimer in the E. amylovora BIM B6-2 mutant as predict by AlphFold3; Figure S6: Schematic representation of a complex Two-Component System (TCS) and Rcs TCS Proteins; Table S1: qPCR assay confirmation on the lack of infection of BIM 6-2 by Erwinia virus φEa46-1A1.
