# Supplementary material for: Multi-Omic Analysis of Bacteriophage-Insensitive Mutants Reveals a Putative Role for the Rcs Two-Component Phosphorelay System in Phage Resistance Development in Erwinia amylovora
Source: Viruses. 2025 Nov 9;17(11):1487. doi: 10.3390/v17111487 (PMC12656757; doi:10.3390/v17111487)
Supplement: Supplementary file 1 [file viruses-17-01487-s001.zip › viruses-3922012-supplementary.pdf]

Supplementary Data

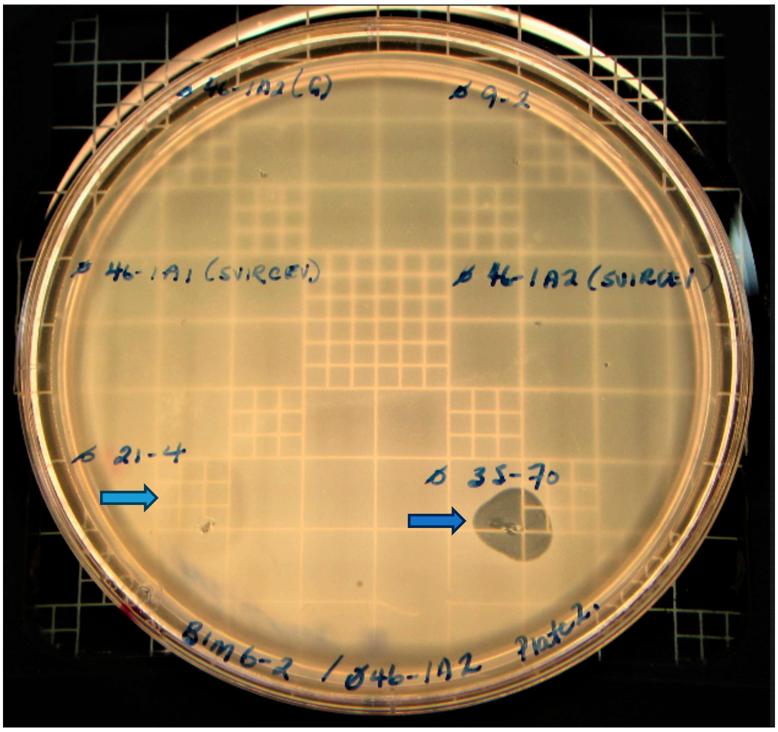

Figure S1. Soft agar overlay with BIM 6-2 as host and spot tests with 6 *Erwinia* phages (clockwise) φEa9-2, φEa46-1A2 (Svircev), φEa35-70, φEa21-4, φEa46-1A1(Svircev) and φEa46-1A2 (G). Clear spots (blue arrows) indicating productive phage infection were produced by myovirus phages φEa21-4 and φEa35-70. All podoviruses (φEa9-2, Ea46-1A, φEa46-1A2) demonstrate non-productive phage infections as indicated by absence of spots. Spot test of 6 *Erwinia* phage against *E. amylovora* EaD7 (positive control) as host produce clear spots, indicating productive infection (Photo not shown). Brackets adjacent to phage names indicate source of phage lysate (Svircev and G, Guelph laboratories).

Table S1. qPCR assay confirmation on the lack of infection of BIM 6-2 by *Erwinia* virus φEa46-1A1.

Treatment	Replicate		*Erwinia* Cy5[f]		Podovirus FAM[g]	
			Ct	Concentration (copies/ml)[h]	Ct	Concentration (copies/ml)
Positive control[a]		STD 10^11	10.3	1.1E+11	12.6	1.1E+11
		STD 10^8	21.1	7.7E+07	23.4	8.2E+07
		STD 10^5	30.7	1.1E+05	33.3	1.1E+05
NTC[b]			No Ct	-	No Ct	-
BIM 6-2[c]	1[d]	1[e]	16.0	2.3E+09	No Ct	-
		2	14.0	9.2E+09	No Ct	-
		3	15.3	3.7E+09	No Ct	-
		4	16.1	2.3E+09	No Ct	-
		5	17.0	1.2E+09	No Ct	-
	2	1	18.1	7.7E+08	No Ct	-
		2	18.3	1.0E+09	No Ct	-
		3	17.3	1.3E+09	No Ct	-
		4	18.0	1.5E+09	No Ct	-
		5	16.6	1.8E+09	No Ct	-
	3	1	17.3	7.7E+08	No Ct	-
		2	16.3	1.0E+09	No Ct	-
		3	16.4	1.3E+09	No Ct	-
		4	15.4	1.5E+09	No Ct	-
		5	16.2	1.8E+09	No Ct	-

[a] *E. amylovora* D7 and *Erwinia* phage φEa46-1A1 (genomically identical to φEa46-1A2). [b] No Template Control. [c] BIM 6-2. [d] Biological replicates. [e] Technical replicates, inoculated with phage φEa46-1A1 at 10^6 PFU/ml. [f,g] Plasmid standard pTotalStdA (Gayder et.al. 2019) for quantitative PCR used to quantify *Erwinia amylovora* and *Erwinia* podovirus (STS3). [h] Concentration of DNA measured in copies/ml.

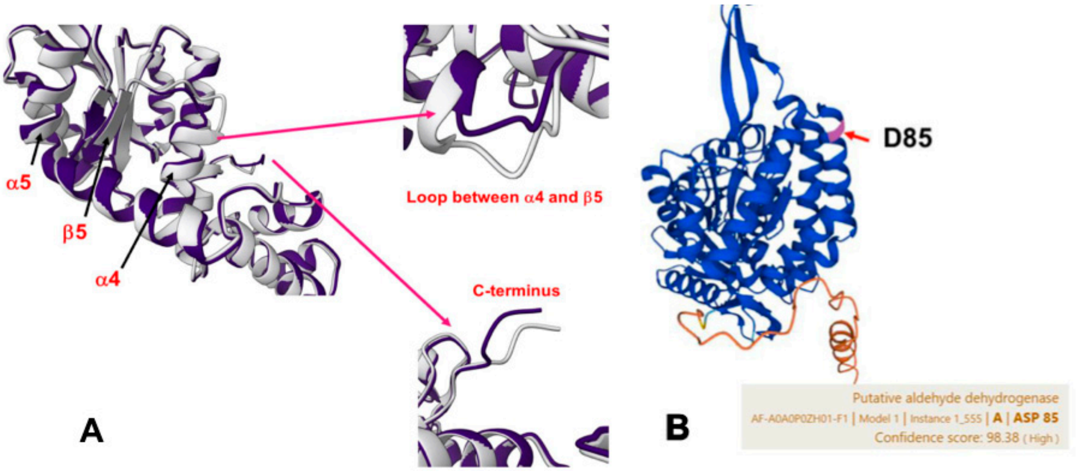

Figure S2. Predicted structural modification caused by the *E. amylovora* BIM B6-2 mutations. A) Superimposition of the predicted RcsB structures from wildtype (accession #: A0A831EKB6) (blue) and BIM B6-2 (gray), as predicted using AlphFold3. Structural element α4-β5-α5 is shown where the LD amino acid insertion in the loop between α4-β5 and the altered C-terminus compared with the wildtype structure (see right insets). B) Predicted folding of the putative *Erwinia* aldehyde dehydrogenase protein (Accession number: A0A0P0ZH01) and the position of the D85>V85 mutation in an α-helix is identified by a red arrow. .

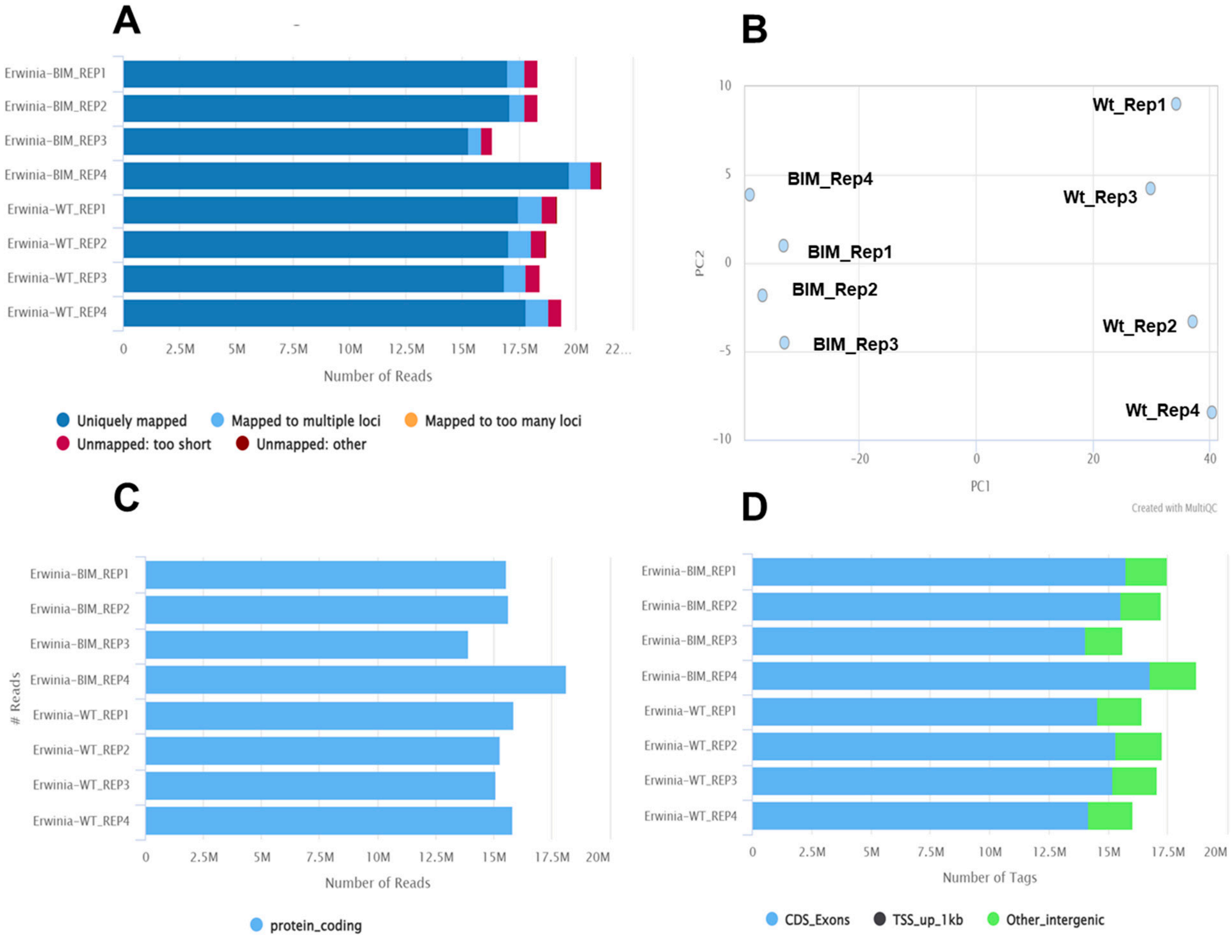

Figure S3. Quality Control Analysis of the Transcriptomic Data of the *E. amylovora* Strains. A) This graph shows the number of nucleotides aligned to

the wildtype reference genome per replicates using the STAR alignment program. The uniquely mapped data (dark blue) is a strong indicator of the good quality of the sequence preparation steps. B) PCA analysis using DSeq2 of the sequenced wildtype and BIM B6-2 replicates. The clustering of the replicates closer to each other compared to the other strain indicates the significant gene expression differences between the two strains. C) The depicted number of protein coding sequences from the generated RNA Seq data for each replicate. D) The exon coding regions (in nucleotide) compared to other parts of the sequence are depicted. The number of exon nucleotides reported in this graph corresponds well with panel C; thereby indicating the good quality of the prepared RNA library and sequenced data.

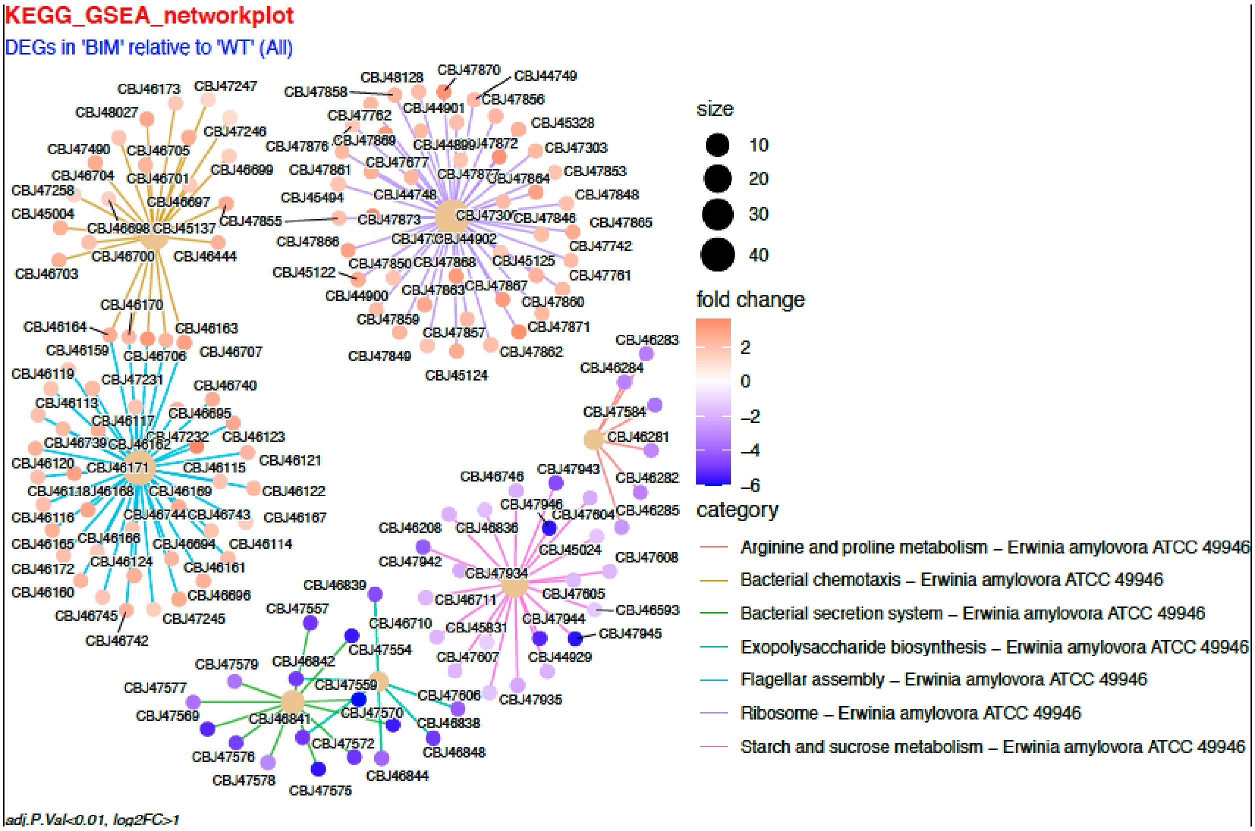

Figure S4. Metabolic Changes Due to the Transcriptomic Data of the *E. amylovora* Strains. The metabolic changes in different pathways that related to the transcriptomics changes in B6-2 mutant compared to that for EaD7 parent strain.

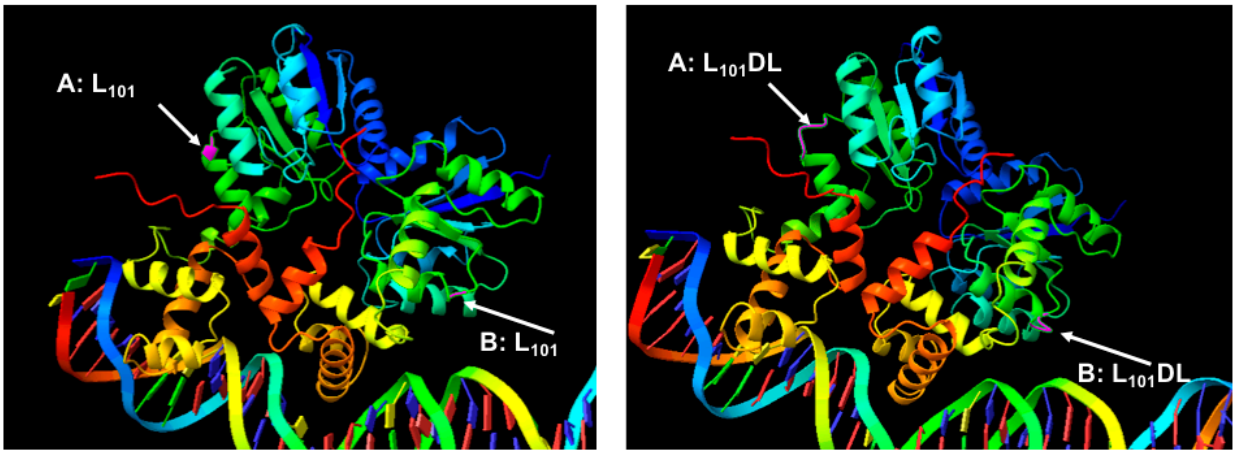

Figure S5. Conformational changes in RcsB homodimer in the *E. amylovora* BIM B6-2 mutant as predict by AlphFold3. A) The predicted wildtype (accession #: A0A831EKB6) RcsB homodimer structure that binds to the RcsAB DNA box as predicted using AlphFold3. Arrows indicated the position of the L_{101} amino acids in both subunits, A&B. B) The predicted B6-2 mutant RcsB homodimer structure that binds to the RcsAB DNA box as predicted using AlphFold3. Arrows indicated the position of the $L_{101}D_{102}$ amino acids insertion in the loop that separate between α4-β5 in both subunits, A&B.

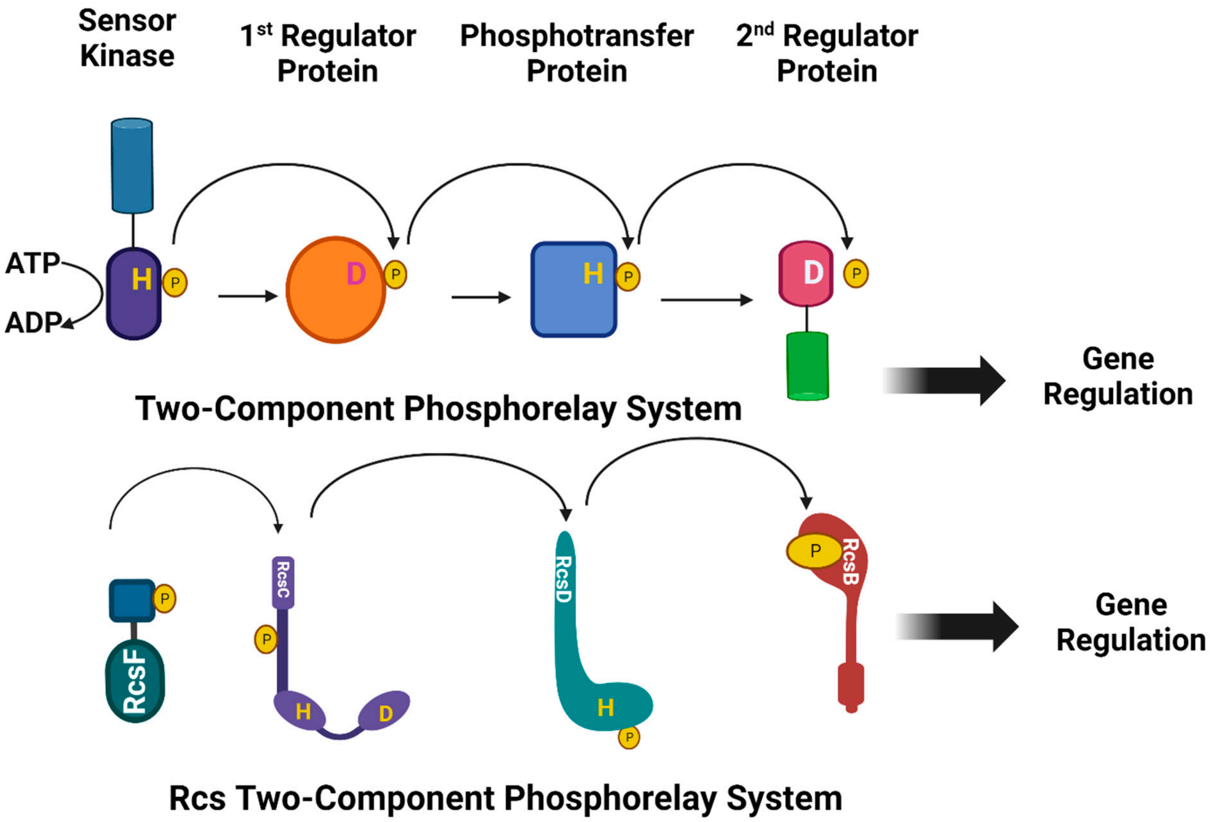

Figure S6. Schematic representation of a complex Two-Component System (TCS) and Rcs TCS Proteins. Upper panel for general TCS proteins. Signal perception and autophosphorylation is the first step in the TCS activation process. It takes place by a sensor kinase protein, which is the signal perception protein and is autophosphorylated from an ATP molecule at a conserved His

(H) residue. The phosphorylated sensor kinase interacts with the first regulator protein, which will receive the phosphoryl group on a conserved aspartate (D) residue from the sensor kinase. Then, the first regulator protein will catalyze the phosphoryl transfer to a conserved His (H) residue in the phosphotransfer protein and ultimately to the second regulator, which will be phosphorylated at a conserved aspartate (D) residue. The phosphorylated second regulator will regulate the gene expression of its target by binding to a specific promoter/DNA Box. In the second panel, the Rcs proteins are depicted in the order of phosphoryl transfer that leads to gene regulation underneath of the protein in the general TCS schematic for which they are predicted to have a similar function.
